# Collective Efficacy in Sports and Physical Activities: Perceived Emotional Synchrony and Shared Flow

**DOI:** 10.3389/fpsyg.2015.01960

**Published:** 2016-01-05

**Authors:** Larraitz N. Zumeta, Xavier Oriol, Saioa Telletxea, Alberto Amutio, Nekane Basabe

**Affiliations:** ^1^Department of Social Psychology and Methodology of the Behavioral Sciences, Faculty of Psychology, University of the Basque CountrySan Sebastian, Spain; ^2^Department of Management and Public Policy, University of Santiago de ChileTalca, Chile

**Keywords:** in-group identification, identity fusion, perceived emotional synchrony, shared flow, collective efficacy, sport and physical activity

## Abstract

This cross-sectional study analyzes the relationship between collective efficacy and two psychosocial processes involved in collective sport-physical activities. It argues that in-group identification and fusion with the group will affect collective efficacy (CE). A sample of 276 university students answered different scales regarding their participation in collective physical and sport activities. Multiple-mediation analyses showed that shared flow and perceived emotional synchrony mediate the relationship between in-group identification and CE, whereas the relationship between identity fusion and CE was only mediated by perceived emotional synchrony. Results suggest that both psychosocial processes explain the positive effects of in-group identification and identity fusion with the group in collective efficacy. Specifically, the role of perceived emotional synchrony in explaining the positive effects of participation in collective sport-physical activities is underlined. In sum, this study highlights the utility of collective actions and social identities to explain the psychosocial processes related to collective efficacy in physical and sports activities. Finally, practical implications are discussed.

## Introduction

During the past years, interest regarding the concept of efficacy from a collective point of view has grown. Collective efficacy represents one of the most studied psychosocial constructs given its implications for performance (Myers et al., 2004), and was initially defined as “the shared perception of a group of its efficacy to perform a behavior and to organize and execute the actions required to reach certain levels of achievement” (Bandura, 1997, p. 447; Martínez et al., 2011). Thus, collective efficacy is a process involving behaviors and interactions observed among members of the group.

Self-efficacy beliefs are related to optimal experiences. In this sense, students showing high self-efficacy beliefs report more flow and, at the same time, teacher's self-efficacy has a reciprocal influence on their optimal experiences over time (Salanova et al., 2014). Different investigations have focused on explaining what variables can predict collective efficacy, including social cohesion and commitment among team members (Beauchamp, 2007; Hampson and Jowett, 2014). Perceived collective efficacy could be an important predictor of performance in different collective settings, such as collective sports (Carron et al., 2002; Leo et al., 2011; Martínez et al., 2011), workgroups (Salanova et al., 2014) and also in political actions (van Zomeren et al., 2004, 2008) or social rituals (Páez et al., 2015).

Due to the role that group processes can play in improving efficacy and performance in collective sports, the general goal of this article was to analyze the relationship of in-group identification and identity fusion of participants in physical and sports activities with their perceived collective efficacy. To achieve this aim, it is important to study the collective processes developed during such activities. Thus, several studies show that gatherings or collective activities strengthen shared emotions and improve positive social beliefs and social cohesion (Rimé et al., 2010; Páez et al., 2015). This study will shed some light over this question.

## Conceptual framework

### In-group identification and identity fusion

In recent years there has emerged a new perspective that focuses on the study of social identity in the collective action (van Zomeren et al., 2008; Thomas et al., 2009). This point of view has shown interest in the individual cognitive changes resulting from social identification. There is evidence regarding the benefits occurring within a group when co-members become part of the collective ego. According to several studies, the extent to which individuals identify with a group may influence their social perception (Ashmore et al., 2004; van Bavel and Cunningham, 2012), involving an individual cognitive change of all members who are part of that group or collectivity. In this sense, a series of experimental studies and interviews have shown how shared in-group membership may enable intra-group trust, more cooperation and comfort in close physical proximity, and a decrease in stress (Drury et al., 2009; Novelli et al., 2010). Leach et al. (2008) integrate the classic and contemporary thinking and have proposed an in-group identification construct that identifies five inter-correlated components: individual self-stereotyping, in-group homogeneity, satisfaction, solidarity, and centrality. This approach has shown construct and predictive validity.

Furthermore, in the last years the concept of fusion of identity with a group has been introduced. It refers to feelings of oneness with the group along with the experience of highly permeable borders between the personal and the social self. Such porous borders encourage people to channel their personal agency into group behavior, raising the possibility that the personal and social self will combine synergistically to motivate pro-group behavior (Gómez et al., 2011; Swann et al., 2012). For instance, the members of a group who declare feeling part of a collective identity feel more identified with the group and have a higher performance than the individuals who report not experiencing high levels of fusion with the group (Swann et al., 2004; Gómez et al., 2011). From this perspective, identity fusion and in-group identification are two distinct aspects: Identity fusion occurs when social identity becomes an essential component of our personal self-concept (Swann et al., 2012), whereas individuals identified with the group share certain prototypical features with other group members that are not essential to their individual personal identities (Gómez et al., 2011). So, when a person is identified with a group, this subject is not relating to other members as a unique individual person, but simply as an anonymous member of the same social category who share the prototypical beliefs, practices, and values of the group (Whitehouse and Lanman, 2014, p. 678).

In sports literature there are not too many studies in which in-group identification or identity fusion with the group have been analyzed (Bruner et al., 2014). Many of the conducted studies were based on the influence that social sport settings have in prosocial and antisocial behaviors (Boardley and Kavussanu, 2010; Rutten et al., 2011), and others on the analysis of the relationship between group cohesion and collective efficacy and performance (e.g., Carron et al., 2002; Leo et al., 2011).

### Shared flow and perceived emotional synchrony as mediating variables

One of the important aspects to take into consideration, both in in-group identification and in identity fusion is the relationship and emotional transformations that take place within groups. When group activities take place, emotions are often shared among the members through social interactions, resulting in a strong emotional contagion. In turn, positive emotional contagion among group members improves cooperation and increased perceived task performance (Barsade, 2002). Also, Lumsden et al. (2014) reported that interpersonal synchrony compared to asynchronous movements elicited in participants more self-esteem, feelings of social connection, and self-other overlap with their partner.

Collective emotional behavior is an interesting phenomenon. In particular, seemingly relevant investigations are the ones aimed at observing social and cultural emotional integration and understanding how they occur and what the convergent synchrony of the affective response implies (von Scheve and Ismer, 2013). According to Kessler and Hollbach (2005) group emotions take place in collective situations where the experienced identities result from being part of a group.

In the early Twentieth century, Durkheim (1912) studied the consequences of mass collective activities. He believed that collective activities implied a collective effervescence that favors an increase of the generated emotional reaction where the subjects, therefore, experience emotions in a joint form. This type of shared emotional experience may imply a mimetic behavior as a result of interacting with other members of the group (Chartrand and Lakin, 2013). The effervescence that develops in a collective gathering can be described as a multifaceted process of social synchronization: participants converge together in special spaces and at particular times; their attention and concentration are focused on the same thing—the core of the social event. Participants display group mimesis or coordinated collective behaviors (shared gestures, shared movements, moving and marching together), thus enacting behavioral synchrony. These behaviors are accompanied by coordinated expressive manifestations (singing together, yelling, saying particular words or sentences, playing music, dancing, etc.) in such a way that every participant's mind, voice and body becomes attuned to the shared emotional state in the group. These preceding elements concur in stimulating participants' emotional arousal in such a way that they will experience and enact similar emotional states. Due to the ease with which emotions are mirrored, shared and spread among people who are co-present, a situation of emotional synchrony develops, thus entailing emotions and perceptions of similarity and unity: “we felt a strong, shared emotion,” “we feel the same, we are the same, we are one” (Collins, 2004; Rossano, 2012; Páez et al., 2013; von Scheve and Ismer, 2013). What is essential is that individuals are gathered together, that common feelings are felt and that they are expressed in common acts. Group members need to be in communion, to be united in the same mind and in the same action (Páez et al., 2015).

It seems then that collective activities, especially group activities, involve situations of emotional synchrony that facilitate the perception of likeness with others, emotional sharing and unity (Rimé et al., 2010; von Scheve and Ismer, 2013), and also performance (Barsade, 2002). Specifically, some recent studies showed that collective emotional gatherings strengthen social identification, social integration, enhance personal and collective self-esteem and positive affect, and positive shared beliefs among participants, through processes of perceived emotional synchrony with others. Participation in symbolic collective gatherings also reinforces fusion of identity or the overlapping of self and others when perceived emotional synchrony is high (Páez et al., 2015). In a similar vein, Whitehouse and Lanman (2014) showed the relationship between rituals and two particular forms of social cohesion: group identification and identity fusion.

Additionally, one of the most studied aspects regarding sports performance is the concept of flow, defined as a deeply rewarding and optimal experience characterized by an intense focus on a certain activity to the point of being totally absorbed by it and the exclusion of all other thoughts and emotions. Within the sports area, different studies on the role of flow in sports performance were carried out in the 90's (Jackson and Marsh, 1996). From these first investigations, flow was considered a key construct to understand the positive experiences in sports, since the experience of flow itself facilitates entering into a state of total concentration during performance (Jackson and Csíkszentmihályi, 2002). In this sense, different studies on various sports showed that athletes who experienced flow while practicing sports tended to enjoy more, derive more satisfaction, and display more concentration and control over their performance (Csíkszentmihályi, 1990; Jackson and Csíkszentmihályi, 2002; Moradi et al., 2014). Flow seems particularly relevant for elite athletes competing at the highest levels under the most intense pressure and experiencing a more rewarding game and, ultimately, showing a very high impact on success (Nicholls et al., 2005).

The concept of flow has usually been studied from an individual point of view within the sports field (Jackson and Csíkszentmihályi, 2002; Jackson and Kimiecik, 2008). However, recent research highlights the importance of experiencing shared flow during physical activity. Csíkszentmihályi (1990) considered collective gatherings or rituals as “affordances that a society offers to its members in order to allow them to meet optimal experiences under socially desirable forms” (p. 432). In this sense Durkheim's concept of collective effervescence and the notion of Csíkszentmihályi's flow are convergent. During optimal experiences, particularly collective ones, participants transcend their ego, get involved in a more complex action system, and the individual feels one with the group he/she acts with (Csíkszentmihályi and Csíkszentmihályi, 1998). In the same line, in Walker's study (2010) athletes revealed that collective flow was more intense and rewarding than individual flow, allowing them experience more enjoyment during sports. Thus, shared flow involves optimal collective experiences during physical and sports activities, where all members of the group experience the same sensation of being absorbed by the activity, while the synchrony of movements and shared emotions increase the perceived collective efficacy.

In a recent study conducted by Schiepe-Tiska and Engerser (2011), they interviewed athletes and concluded that synchronized movements and a shared focus on attentiveness among peers were essential elements to experience flow in the competition. Current experimental studies have found significantly greater group cooperation when synchrony and shared intentionality were combined (Reddish et al., 2013). Therefore, another aim of this investigation was to establish the specific contribution of each of these psychosocial processes, flow experience and perceived emotional synchrony, in promoting collective efficacy in group physical and sport activities. This study will enable us to analyze this issue.

## Objectives and hypothesis

The main goal of this study was to analyze the predictor variables of collective efficacy (in-group identification and identity fusion) and to investigate the mediating effects produced by collective processes (shared flow and perceived emotional synchrony with others) while in-group sport activity takes place. We proposed that these types of variables would have a vital impact on the sense of perceived collective efficacy.

**Figure 1:**
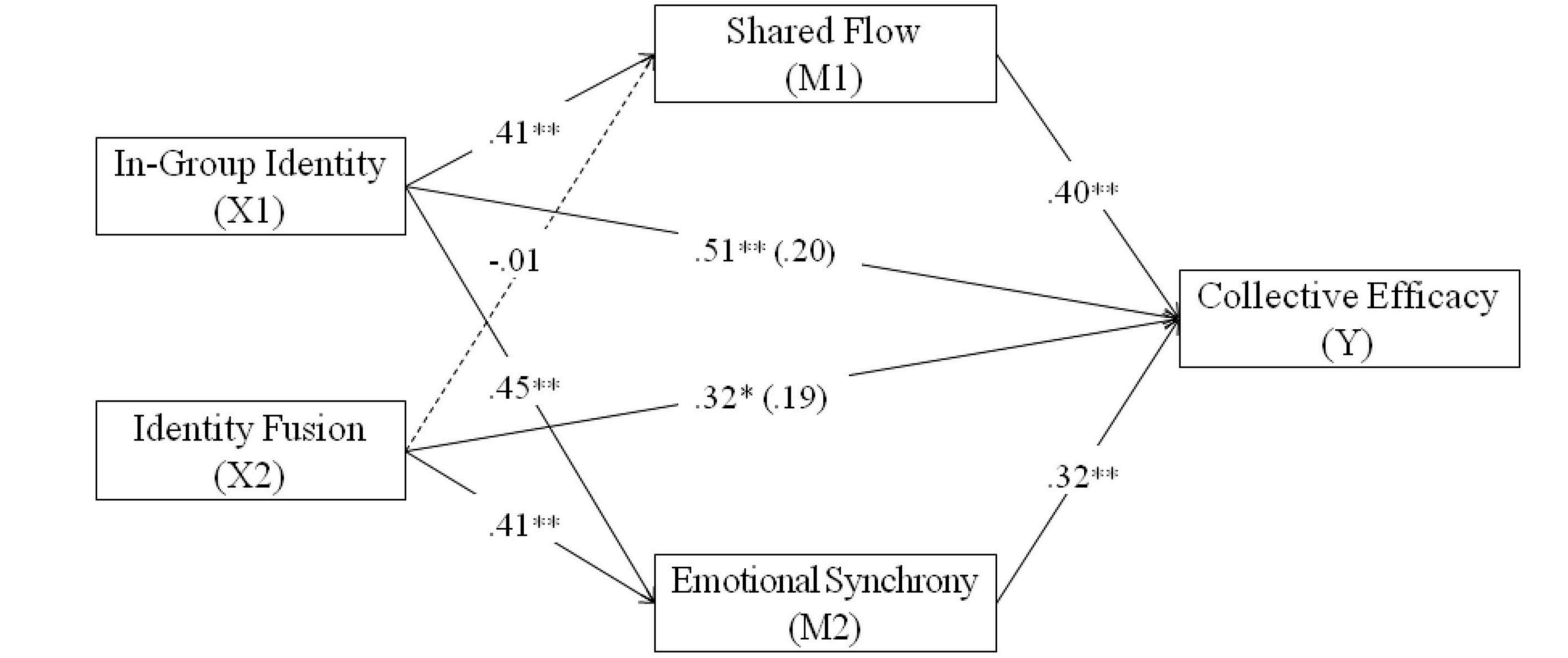
**Multiple-mediation model for collective efficacy mediated by shared flow and perceived emotional synchrony**. ^**^p < 0.01; ^*^p < 0.05.

Using a multiple-mediation model, the following effects were examined: (a) The indirect or mediating effect of shared flow in the relationship between in-group identification and collective efficacy; (b) The mediating effect of shared flow in the relation between identity fusion and collective efficacy; (c) The indirect or mediating effect of perceived emotional synchrony in the relationship between in-group identification and collective efficacy; and (d) The mediating effect of perceived emotional synchrony in the relation between identity fusion and collective efficacy. The multiple-mediation model is shown in Figure 1.

The following hypotheses were formulated: (H1) In-group identification and identity fusion will have direct effects on collective efficacy; (H2) Shared flow will mediate the relation between in-group identification and collective efficacy (H2a), and between identity fusion with the group and collective efficacy (H2b); (H3) Perceived emotional synchrony will mediate the relationship between in-group identification and collective efficacy (H3a), and between identity fusion and collective efficacy (H3b).

## Materials and methods

### Participants

The convenience sample consisted of 276 volunteer university students (71% women, 29% men) between 19 and 30 years of age (*M* = 21, *SD* = 2.28). All participants practiced some type of collective physical activity and sports every week. It is important to note that this research was intended not just to collect a sample of participants in competitive sports, but it also included group physical activities in general (leaving aside daily physical activities, such as domestic and work-related activities).

### Procedure

University students (Physical, Education Sports, and Social Sciences students) voluntarily participated in the study. Once informed, participants signed their written consent and the registered data was alphanumerically coded, ensuring anonymity, in order to comply with the Personal Data Protection Act by the Ethics Committee for Research related to Human Beings (CEISH). Questionnaires were completed by the students in the classrooms. Data collection was conducted over 2 months in 2013.

All participants had to specify the type of collective physical activity they had recently practiced with other people. The physical and sports activities were classified by agreement between the main researchers of this investigation into four categories: (1) collective sports federated, (2) non-federated (e.g., football, basketball, handball, rugby, volleyball, water polo, rowing, regatta, rhythmic gymnastics, karate, and Basque ball), (3) Physical activities or sports in group (e.g., aerobics, indoor cycling, different types of dance -Basque, oriental, jazz-, kick boxing, different types of gymnastics, running, and climbing), and (4) Recreational physical activities involving other people (e.g., outdoors activities, such as, hiking, running, ski, walking, trekking, skating, cycling, horse riding, dancing, and extreme sports). At the same time, these categories were grouped into two types: team sports (1 and 2) and social exercise (3 and 4).The time required to complete the questionnaires was between 30 and 40 min.

### Measures

#### The in-group identification scale

It was developed by Leach et al. (2008). A Spanish version was used. It is comprised of 14 items measuring Solidarity (“I am in solidarity with the people in my group”); Satisfaction (“It is nice to be part of my group”); Centrality (“Belonging to my group is an important part of my identity”); Typical Similarity (“I am similar to a typical person of my group”); and Integration (“The individuals in my group have a lot in common with each other”). The range of response was seven points from 1 (*Totally disagree*) to 7 (*Totally agree*). This version scale has been applied in several studies within the context of this study, showing reliability and structural validity with one common dimension (Bobowik et al., 2013). A factorial analysis was conducted imposing one single factor, and explaining 55.43% of the total variance [KMO = 0.93, $\chi_{\left(91\right)}^{2}=$ 2844.801, *p* < 0.001]. Cronbach's α = 0.94.

#### Verbal fusion scale

It is an adaptation of the Spanish version created by Gómez et al. (2011). It consists of seven items that measure the feeling of fusion with the group (e.g., “I feel a strong emotional bond with my group”; “In my group I feel as part of a whole”), with a rank of response from 1 (*Totally disagree*) to 7 (*Totally agree*). An exploratory factorial analysis resulted in a unidimensional structure, which explained the 68.13% of the total variance [KMO = 0.89, $\chi_{\left(21\right)}^{2}=$ 1430.21; *p* < 0.001]. Cronbach's α = 0.92.

#### Shared flow scale

The scale was originally derived from the Spanish version of Jackson and Marsh's Dispositional Flow Scale (1996) and its adaptation by García et al. (2008). It has been reformulated by Zumeta et al. (2013) to explore the optimal shared group experiences (e.g., “We knew that our capabilities would enable us to face the challenge posed to us”; “We were totally focused on what we were doing”). The scale comprises 27 items distributed in nine dimensions: (1) Balance between challenge and skill; (2) Action-awareness merging; (3) Clear proximal goals; (4) Unambiguous and direct feedback; (5) Focused concentration on the current activity; (6) Sense of control over ones actions; (7) Loss of self-consciousness; (8) Loss of time awareness or time acceleration; (9) Autotelic experience; and a global second order factor encompassing all dimensions. In this investigation the model consisting of a one-dimensional structure was selected because components of shared flow converge in a unique dimension, showing a good construct validity and internal consistency. CFA for this model revealed the following data: $\chi_{\left(315\right)}^{2}=$ 637.93, p < 0.001, *CFI* = 0.92, *TLI* = 0.906, RMSEA = 0.06 (90% CI [0.05, 0.07]). The rank of response was from 1 (*Totally disagree)* to 7 (*Totally agree*). Cronbach's α was 0.93.

#### Perceived emotional synchrony

This scale was developed by Páez et al. (2015) to assess the degree in which participants experienced an emotional contagion or shared emotion condition (e.g., “We felt a strong, shared emotion”), and perceived synchrony with others (e.g., “We all acted as if we were one person”). The scale is comprised of 16 items with a rank of response from 1 (*Not at all)* to 7 (*Very much*). An exploratory factorial analysis showed a one-factor structure, which explained the 72.15% of the total variance (KMO = 0.97, $\chi_{\left(120\right)}^{2}=$ 4683.08; *p* < 0.001). Cronbach's α = 0.97.

#### Collective efficacy questionnaire for sports scale

A short, 4-item version was adapted from the CEQS-Collective Efficacy Questionnaire for Sports (Martínez et al., 2011), assessing respondents' perceived efficacy of the group with which they performed a sport-physical activity (i.e., “Show more abilities than other groups”; “Effectively prepared for the activities”; “Ability to overcome distractions”; and “Performing the activity better than other groups”), and with a rank of response of 11 points (0 = *not at all* to 10 = *very much*). A previous study conducted by Páez et al. (2015) used this brief scale and an adequate factorial structure was observed. In the current study, an exploratory factorial analysis showed a one-dimensional structure, which explained the 75.91% of the total variance (KMO = 0.78, $\chi_{\left(6\right)}^{2}=$ 667.16; *p* < 0.001). Cronbach's α was 0.89 for the four items used.

### Design and analyses

This is a single-group, cross-sectional observational study based on different scales applied to university students. Statistical descriptions and bivariate correlations were calculated through the SPSS 20.0. To analyze the effects of shared flow and emotional perceived synchrony (multiple mediation) in relation to collective efficacy, the bootstrap procedure proposed by Preacher and Hayes (2004) was applied through the SPSS macro MEDIATE for models with multiple independent variables. This analysis estimates the indirect effect, standard errors and confidence intervals. Non-parametric bootstrapping procedure was used with 5000 repetitions, 95% confidence intervals, to verify the mediation effect of both variables. The indirect effect is significant if the confidence interval does not exceed zero value (Preacher and Hayes, 2004; Hayes, 2013). To test the indirect effects and calculate the effect size we use the PROCESS macro that calculated the ratio of the indirect effect to the total effect, that is to say, how much of the effect of X on Y operates indirectly through M (i.e., the proportion of the total effect that is mediated), and the ratio of the indirect effect to the direct effect (R_M_). So, if R_M_ > 1, then the indirect effect is larger than the direct effect, whereas if R_M_ < 1, the indirect effect is smaller than the direct effect (Hayes, 2013, p. 189). Also Kappa-square (κ^2^), i.e., the ratio of the indirect effect to its maximum possible value in the data (it is bound between 0 and 1), was calculated. The level of significance used was *p* < 0.05. Finally, in order to estimate the moderated mediation models, with tasks as the moderator (W), Hayes procedure was applied (2013). Conditional indirect effects of Xi (in-group identification and fusion with the group) on Y (collective efficacy) through Mi (shared flow and perceived emotional synchrony) as a function of a moderator W, (e.g., type of task, a categorical variable composed by team sports vs. social exercise), were calculated. Moderated mediation models were estimated using macro PROCESS by specifying model seven reported by Hayes (2013, p. 447).

## Results

Analyses of central tendency show that all variables have average values above the scale midpoint (3.50 for all variables, except collective efficacy, with a scale midpoint of 5.50). All the studied variables reached high values during the group physical and sport activities (see Table 1). High levels for shared flow and group identification were especially reported. Table 1 shows the statistical descriptions, average, and standard deviation for each of the variables, as well as the bivariate correlations among them. Missing values for each variable reached values below 5%.

**Table 1:** **Means, standard deviations and correlations among variables**.

**Variable**	***M***	***SD***	**1**	**2**	**3**	**4**	**5**
1. In-group identification	5.14	1.21	–				
2. Identity fusion	4.68	1.35	0.85	–			
3. Shared flow	5.31	0.83	0.54	0.48	–		
4. Emotional synchrony	4.58	1.39	0.71	0.73	0.64	–	
5. Collective efficacy	6.45	1.77	0.55	0.51	0.50	0.56	–

*Note, All p < 0.001 (bivariate)*.

As can be seen, all variables correlate significantly and positively. The size of the correlation coefficients indicates the presence of high-medium positive associations between the studied variables. The rank of correlations fluctuates from the lowest correlation between identity fusion and shared flow, *r* = 0.48, *p* < 0.001 to the highest found between in-group identification and identity fusion with the group, *r* = 0.85, *p* < 0.001.

Participants selected the following physical and sport activities performed during leisure time: (1) competitive federated collective or team sports (24.2%); (2) non-federated collective sports (10.8%); (3) physical activities or sports in group (14.1%); and (4) recreational physical activities or free time with other people (50.9%). Overall, 35% performed team sports and 65% social exercise activities; more men choose team sports (66%) while women preferred social activities (77%).

Results showed no significant differences between different physical-sport activities in the procedural variables of the study, shared flow [*F*_(3, 261)_ = 0.218, *p* < 0.884] and perceived emotional synchrony [*F*_(3, 261)_ = 1.56, *p* < 0.198]. However, there were some differences between types of activities for in-group identification [*F*_(3, 264)_ = 4.44, *p* = 0.005], identity fusion [*F*_(3, 267)_ = 6.68, *p* = 0.001], and collective efficacy [*F*_(3, 270)_ = 4.67, *p* = 0.003]. Concretely, *post-hoc* Scheffé contrasts showed that in-group identification was higher for federated team sports (1) (*M* = 5.33, *SE* = 1.06) and recreational physical activities (4) (*M* = 5.24, *SE* = 1.13) than for physical activities or sports in group (3) (*M* = 4.51, *SE* = 1.55; *p* = 0.011, *p* = 0.012, respectively). Similar differences were obtained for identity fusion between types of activities: federated team sports (1) (*M* = 4.96, *SE* = 1.30) and recreational physical activities (4) (*M* = 4.79, *SE* = 1.17) as compared to physical activities or sports in group (3) (*M* = 3.84, *SE* = 1.70; *p* = 0.011, *p* < 0.001, respectively). Finally, participants in federated team sports reported higher collective efficacy (1) (*M* = 6.98, *SE* = 1.60) than participants in recreational physical activities (4) (*M* = 6.18, *SE* = 1.75, *p* = 0.024).

### Mediation analysis: the role of shared flow and perceived emotional synchrony with others

Mediation analysis (Figure 1) was used to study if the degree of in-group identification (X1) and identity fusion (X2) while performing a physical and sport group activity showed significant indirect effects on collective efficacy (Y) through shared flow (M1) and perceived emotional synchrony (M2).

Regression analysis confirmed a significant effect of in-group identification on shared flow (*B* = 0.41, *SE* = 0.07, *t* = 5.63, *p* < 0.001) and perceived emotional synchrony (*B* = 0.45, *SE* = 0.10, *t* = 4.60, *p* < 0.001). However, in the case of identity fusion with the group, no significant effect was found on shared flow (*B* = −0.01, *SE* = 0.06, *t* = −0.14, *p* = 0.891) although a significant effect was found on perceived emotional synchrony (*B* = 0.41, *SE* = 0.08, *t* = 4.91, *p* < 0.001). Both, shared flow (*B* = 0.40, *SE* = 0.15, *t* = 2.73, *p* = 0.004) and perceived emotional synchrony (*B* = 0.32, *SE* = 0.11, *t* = 2.93, *p* = 0.004) were significant and positive predictors of collective efficacy. Finally, a significant effect of in-group identification (*B* = 0.51, *SE* = 0.16, *t* = 3.20, *p* = 0.001) and identity fusion (*B* = 0.32, *SE* = 0.14, *t* = 2.33, *p* = 0.021) on collective efficacy was found, confirmed by the total “accumulated” effect of the test [*F*_(2, 245)_ = 57.04, *p* < 0.001], which was also significant. Furthermore, once the mediating variables, shared flow and perceived emotional synchrony were introduced, it was observed that the direct effects of in-group identification and identity fusion with the group as predictor variables upon collective efficacy were not statistically significant (*B* = 0.20, *SE* = 0.16, *t* = 1.24, *p* = 0.214 y *B* = 0.19, *SE* = 0.14, *t* = 1.38, *p* = 0.168), therefore, the result is a total mediation.

In addition, there was a significant indirect effect of in-group identification on collective efficacy through shared flow (*B* = 0.16, *SE* = 0.07, 95% CI [0.033, 0.315]), and also through perceived emotional synchrony (*B* = 0.14, *SE* = 0.06, 95% CI [0.036, 0.283]). In other words, in-group identification increased perceived collective efficacy through shared flow and perceived emotional synchrony. The mediation analysis confirmed that this relationship was explained by these two processes, given the significance of the indirect effects. On the other hand, the indirect effect of identity fusion with the group on collective efficacy through shared flow was not significant. (*B* = −0.003, *SE* = 0.02, 95% [−0.064, 0.562]), although it was significant through perceived emotional synchrony (*B* = 0.13, *SE* = 0.05, 95% [0.035, 0.264]). This indicates that the level of fusion with the group increases perceived collective efficacy through perceived emotional synchrony, and not through shared flow.

To test the indirect effects and size effects we use the ratios between effects and the kappa-square index (Hayes, 2013). Results show the ratio of the indirect effect to the total effect of in-group identification on collective efficacy (*B* = 0.56, *SE* = 0.13, 95% [0.343, 0.881]), composed of the perceived emotional synchrony ratio (*B* = 0.38, *SE* = 0.12, 95% [0.166, 0.656]), and the shared flow ratio (*B* = 0.17, *SE* = 0.08, 95% [0.030, 0.356]). Also, the indirect effects are larger than the direct effects (RM = 1.30). Kappa-square −κ^2^− indicates a medium-low effect size for perceived emotional synchrony (κ^2^ = 0.21, *SE* = 0.05, 95% [0.118, 0.307]) and for shared flow (κ^2^ = 0.15, *SE* = 0.04, 95% [0.080, 0.225]).

Ratios for significant indirect effects of identity fusion were also calculated. The ratio of perceived emotional synchrony indirect effect to the total effect of identity fusion on collective efficacy was *B* = 0.49, *SE* = 0.12, 95% [0.273, 0.761] and the indirect effect was smaller than the direct effect (RM = 0.96). The kappa-square indicates a medium-low effect size for perceived emotional synchrony (κ^2^ = 0.21, *SE* = 0.05, 95% [0.128, 0.305]). To summarize, perceived emotional synchrony showed higher indirect effect size than shared flow upon collective efficacy. The indirect effects were larger than the direct effects for in-group identification, but not in the case of identity fusion where the direct and indirect effects were very similar.

Lastly, to verify if the hypothesized direct and indirect effects were dependent on the type of sport and physical activity performed two moderated mediation models were estimated. Tables 2, 3 present the results of the moderated mediation analyses for collective efficacy by task with direct, indirect, and conditional effects. Conditional direct and indirect effects for each of the values of the moderator variable (0 = team sports vs. 1 = social exercise) were included. As can be seen in Table 2 the interaction effects of in-group identification by task were not statistically significant according to 95% confidence intervals given that they contained the value 0 in all cases. With respect to conditional effects, findings showed that direct and indirect effects of in-group identification on collective efficacy were statistically significant in both of the tasks, that is, team sports and social exercises. In this way, the postulated effects of in-group identification on collective efficacy and also the indirect effects of share flow and emotional synchrony were significant because confidence intervals do not include the 0 value.

**Table 2:** **Moderated mediation model for in-group identification on collective efficacy by task: direct, indirect, and conditional effects**.

**Variable**	***B***	***SE***	***t***	***p***	**LL 95% CI**	**UL 95% CI**
**MEDIATOR VARIABLE MODEL: SHARED FLOW**						
Constant	2.92	0.35	8.21	<0.001	2.224	3.627
In-group identification	0.46	0.06	6.97	<0.001	0.333	0.595
Task	0.47	0.42	1.12	0.260	−0.354	1.300
Interaction_1	−0.09	0.07	−1.16	0.243	−0.248	0.063
**MEDIATOR VARIABLE MODEL: EMOTIONAL SYNCHRONY**						
Constant	0.40	0.50	0.80	0.419	−0.583	1.398
In-group identification	0.80	0.09	8.50	<0.001	0.623	0.993
Task	−0.32	0.59	−0.54	0.587	−1.491	0.846
Interaction_1	0.07	0.11	0.68	0.489	−0.143	0.296
**DEPENDENT VARIABLE MODEL: COLLECTIVE EFFICACY**						
Constant	1.32	0.82	1.59	0.112	−0.310	2.948
Shared flow	0.34	0.14	2.44	0.015	0.067	0.625
Emotional synchrony	0.39	0.10	3.89	<0.001	0.192	0.587
In-group identification	0.38	0.16	2.45	0.014	0.077	0.698
Task	−0.25	0.85	−0.29	0.767	−1.945	1.436
Interaction_2	−0.08	0.16	−0.53	0.591	−0.405	0.231
**CONDITIONAL DIRECT EFFECTS AT THE VALUES OF TASK**						
**Task**						
0 = Team sports	0.38	0.15	2.45	0.014	0.077	0.698
1 = Social exercise	0.30	0.11	2.50	0.012	0.064	0.537
**CONDITIONAL INDIRECT EFFECTS AT THE VALUES OF TASK**						
**Shared flow**						
0 = Team sports	0.16	0.07	0.037			0.323
1 = Social exercise	0.12	0.05	0.025			0.255
**Emotional synchrony**						
0 = Team sports	0.31	0.09	0.151			0.529
1 = Social exercise	0.34	0.09	0.159			0.541

*Task: 0 = Team sports vs. 1 = Social exercise*.

*Interaction_1 = Identity fusion × Task; Interaction_2 = Identity fusion × Task*.

*LL, Lower limit; CI, Confidence interval; UL, Upper limit*.

**Table 3:** **Moderated mediation model for identity fusion on collective efficacy by task: direct, indirect, and conditional effects**.

**Variable**	***B***	***SE***	***t***	***p***	**LL 95% CI**	**UL 95% CI**
**MEDIATOR VARIABLE MODEL: SHARED FLOW**						
Constant	3.75	0.29	12.61	<0.001	3.171	4.344
Identity fusion	0.32	0.05	5.52	<0.001	0.209	0.442
Task	0.21	0.35	0.58	0.555	−0.491	0.912
Interaction_1	−0.04	0.07	−0.58	0.558	−0.183	0.099
**MEDIATOR VARIABLE MODEL: EMOTIONAL SYNCHRONY**						
Constant	1.02	0.39	2.63	<0.001	0.261	1.798
Identity fusion	0.74	0.77	9.65	<0.001	0.594	0.899
Task	0.06	0.46	0.14	0.885	−0.852	0.987
Interaction_1	0.05	0.09	0.05	0.952	−0.179	0.191
**DEPENDENT VARIABLE MODEL: COLLECTIVE EFFICACY**						
Constant	1.45	0.73	1.97	0.049	0.012	2.910
Shared flow	0.49	0.13	3.65	<0.001	0.230	0.768
Emotional synchrony	0.32	0.10	3.09	<0.001	0.117	0.527
Identity fusion	0.27	0.13	2.09	0.036	0.017	0.537
Task	−0.74	0.67	−1.09	0.275	−2.082	0.596
Interaction_2	0.01	0.13	0.12	0.899	−0.252	0.287
**CONDITIONAL DIRECT EFFECTS AT THE VALUES OF TASK**						
**Task**						
0 = Team sports	0.27	0.13	2.09	0.036	0.017	0.537
1 = Social exercise	0.29	0.10	2.82	<0.001	0.089	0.500
**CONDITIONAL INDIRECT EFFECTS AT THE VALUES OF TASK**						
**Shared flow**						
0 = Team sports	0.16	0.06	0.064		0.307	
1 = Social exercise	0.14	0.04	0.062		0.249	
**Emotional synchrony**						
0 = Team sports	0.24	0.08	0.080		0.426	
1 = Social exercise	0.24	0.08	0.077		0.426	

*Task: 0 = Team sports vs. 1 = Social exercise*.

*Interaction_1 = Identity fusion × Task; Interaction_2 = Identity fusion × Task*.

*LL, Lower limit; CI, Confidence interval; UL, Upper limit*.

The second moderated mediation model with identity fusion as predictor showed similar results, that is, interaction effects were not significant and direct and indirect effects were significant in both tasks (see Table 3). These results confirm that our hypotheses are applicable to different types of collective sports and physical activities reported by participants in this study and support the notion that fusion and identification with the group that share a collective physical activity is related to positive effects on participants, whether they participate in sports groups or engage in other physical activities.

## Discussion

First, and according to our first hypothesis, we could confirm that both in-group identification and identity fusion with the group showed a total effect on collective efficacy. This result is consistent with the data from other studies on the importance that feeling part of a group and identifying with the other members has on group's performance (Swann et al., 2004; Salanova et al., 2014) and particularly, in this case, on collective efficacy. Likewise, Fransen et al. (2014) observed in a very recent study that team identification showed a predictive effect on collective efficacy in a sample of athletes. In addition, fusion of identity could favor the construction of joint beliefs. In this sense, some research in other areas has shown that the fused persons respond to irrevocable ostracism from other group members by increasing their willingness to sacrifice themselves for the group (Gómez et al., 2011). According to the Social Cognitive Theory (Bandura, 1997, 2001), people share beliefs in their collective power to produce the desired results (collective efficacy beliefs). Our results confirm our consideration about the superiority of group identity, as compared to individual identity, to promote the strengthening of collective efficacy beliefs on the part of group members.

In line with our second hypothesis, we expected shared flow to mediate the relationship between in-group identification and identity fusion on collective efficacy. The results confirm an indirect effect of in-group identification on collective efficacy through shared flow; however, this indirect relation was not found with identity fusion. Therefore, the hypothesis was confirmed in the case of in-group identification, but not for identity fusion in the group. Thus, it is interesting to find that shared flow, an emotion related to optimal, autotelic experiences and which involves pleasurable feelings is related to in-group identification (Rimé et al., 2010; von Scheve and Ismer, 2013). Unlike what happens with individual physical activities, in the collective ones, participants hold a reciprocal relationship between them, which we believe reinforces collective efficacy. In addition, it should be noted that for there to be an increase in these beliefs of efficacy, it is also vital that participants experience cognitive and affective psychological positive states (Amutio et al., 2015a), including shared flow. A recent multi-sample study conducted by Walker (2010) showed that social flow is more enjoyable than solitary flow, thereby supporting the claim that “doing it together is better than doing it alone.”

As discussed above, it should be noted that in the relation between identity fusion and collective efficacy, shared flow does not behave as a mediator. Perhaps, the main reason is that fusion of identity with the group does not entail the experience of positive affection, as it is the case with flow. Some studies showed that fusion with the group implies that someone is willing to sacrifice for the collectivity (Gómez et al., 2011; Swann et al., 2012); therefore, it is not related to the experience of joy, but to an intrinsic interest in the task. Something different happens with in-group identification. Identification exerts a predictive effect on shared flow, and additionally, shared flow is mediating the relationship between in-group identification and collective efficacy. Furthermore, a total effect between shared flow and collective efficacy is observed. The differential effects of these two components of social cohesion, in-group identification and identity fusion, support the point of view that are distinct phenomena (Gómez et al., 2011; Swann et al., 2012).

There are still very few studies focused on shared flow. However, individual flow has been associated with a greater degree of concentration in the task and with an increase in athlete's self-efficacy (Nicholls et al., 2005; Salanova et al., 2014). Athletes who experience situations of flow during the competition tend to enjoy more and have more control over sports performance (Csíkszentmihályi, 1990; Jackson and Csíkszentmihályi, 2002). This fact can be associated with the total effects observed between shared flow and collective efficacy. Data provided by this study confirm some findings obtained so far, which demonstrate the importance of flow among team members for performance improvement in competition (Walker, 2010; Schiepe-Tiska and Engerser, 2011).

Regarding the mediating function of shared flow in the relationship between in-group identification and collective efficacy, the total mediation observed proves the importance of experiencing collective flow during sports and physical activities. Previous studies have demonstrated the influence that group identity has on the athletes' social perception (Ashmore et al., 2004; van Bavel and Cunningham, 2012). Specifically, it seems that in-group identification can be understood as a mode of favoring the self-empowerment of members with the purpose of achieving their collective goals and striving for the social development of the group (Drury and Reicher, 2005). Likewise, a recent study has concluded that shared flow entails empowerment effects, reinforcing collective efficacy (Páez et al., 2015).

In consonance with the results of this study, perceived collective efficacy through in-group identification would be totally mediated by the experience of flow during sports activity. Studies on individual flow in sports have proved that experiencing it while practicing a sport involves more concentration and control over performance (Jackson and Csíkszentmihályi, 2002; Jackson and Kimiecik, 2008). Therefore, it may be considered that experiencing shared flow during the physical-sport activity is essential for group members. Accordingly, those who share a collective identity may perceive themselves as more efficient as a group.

We have proposed that flow experiences promote collective efficacy. However, the cross-sectional nature of the current study limits this conclusion. Recently, a longitudinal and collective study has analyzed the group flow experiences by considering efficacy beliefs as both antecedents and consequences Thus, flow experiences involving positive affects and moods facilitate positive evaluations and, thereby, predict the development of efficacy beliefs. Inversely, it is also plausible that shared beliefs in collective efficacy could influence perceptions of challenges and skills, and in turn lead people to experience flow in collective settings. This research findings confirmed the longitudinal relationship between collective efficacy beliefs and collective flow, suggesting that positive collective experiences (shared flow) are a consequence and also a source of feelings of efficacy (Salanova et al., 2014).

As for our third hypothesis, perceived emotional synchrony has shown an indirect effect on the relationship among in-group identification and identity fusion with collective efficacy. This study shows the crucial role of perceived emotional synchrony to explain the positive effects of participation in collective gatherings, in this case, collective sport-physical activities. These findings confirm the first approaches of Durkeim's collective effervescence (1912) and help to explain much better the role of collective emotions in the perceived efficacy of sports practice. Durkheim considered that collective activities implied that subjects shared the same mood and this evoked a mutual comforting sensation (Rimé et al., 2010). In this sense, our results show that in-group identification and identity fusion are favoring the perceived emotional synchrony of the group members during the sports activity. Thus, in collective emotional situations (Durkheim's emotional communion), the individuals highly fused with the group will experience an increased sensation of collective synchrony resulting in a strong sense of collective efficacy, and this effect is not explained by shared flow. Perceived emotional synchrony implies an emotional contagion between the group members and sharing emotions among them, as well as a perceived synchronic behavior. As several studies have shown, collective behaviors involving movement and emotional synchrony promote perceived similarity with others, emotional sharing, and unity (Rossano, 2012; von Scheve and Ismer, 2013; Páez et al., 2015).

According to the literature, in-group identification and identity fusion involve a higher feeling of cooperation between group members and being more comfortable with physical closeness (Reicher and Haslam, 2006; Novelli et al., 2010), which would be fully associated with perceived emotional synchrony during physical activity and sports. In-group identification and fusion of group members prior to the sports activity may favor an implicit corporal communication, that has been associated with psychosocial functions, such as promoting social cohesion, improving the feelings of connection, and the relationship and cooperation between the interacting individuals (Reddish et al., 2013). The indirect effects observed between in-group identification and identity fusion over collective efficacy would also explain the fact that perceived emotional synchrony between group members would be fully mediating this relationship.

## Concluding remarks

This is the first study to demonstrate the role of perceived emotional synchrony and shared flow in promoting collective efficacy in sports and physical activities. In sports psychology, collective efficacy is a very important construct aimed at enhancing performance in team sports. Within the social and positive psychology areas, collective activities represent opportunities to share positive experiences among team members. This research aims to contribute to increase understanding of this concept by observing how collective processes related to shared flow and perceived emotional synchrony in sports and physical activities are variables that explain and also have a decisive influence on the perceived collective efficacy of groups.

The findings on this study show the predictive relationships of in-group identification and identity fusion with the group on perceived collective efficacy. However, this relation has been mediated by the experience of shared flow, in the case of in-group identification, and by perceived emotional synchrony (both in in-group identification and identity fusion with the group). This outcome suggests, therefore, that shared flow and emotional synchrony are collective processes occurring during collective sports and physical activities, and they substantially influence the perceived efficacy of the group. Particularly, perceived emotional synchrony during the physical-sport activity is a substantial process, enabling athletes to experience a higher collective efficacy, which would directly impact on their performance. Nevertheless, the consequences of collective efficacy on group performance are not analyzed in this study. This question needs to be explored in future studies.

This study emphasizes that sport and physical activities performed in company of others constitute an excellent framework for the study of collective emotional processes. In recent years, there is a growing interest in the study of shared flow (Walker, 2010; Salanova et al., 2014) and collective emotions (Collins, 2004; Páez et al., 2015). Therefore, the relevance and novelty of this study has been to demonstrate the importance of group identification in its different forms (in-group identification and fusion with the group), and the substantial role of perceived emotional synchrony and shared flow as mechanisms that can explain group effects during collective gatherings, including physical activities. These results provide substantial evidence that participation in collective physical activities promotes group identification, creates bonds between people, and bring participants to a stage of emotional synchrony and optimal experience. Our findings are in line with previous studies (Páez et al., 2015) which proved that participation in collective gatherings reinforces collective identity, as well as having positive effects, in this case promoting collective efficacy.

Among the limitations of the present study we should note that data are cross-sectional and retrospective and therefore do not allow for causal claims about the associations between variables. In this sense alternative models will be plausible, for example, the experience of emotional synchrony or shared flow would impact collective efficacy by increasing group identification or identity fusion, or even reciprocal effects between shared flow and collective efficacy. A second limitation is related to the type of participants (students and predominantly women). Future studies will be able to use different types of samples (e.g., athletes) and more gender-balanced samples that could add more heterogeneity in sports and physical activities performed collectively.

Despite the limitations, practical applications of these results are important. To elaborate, collective management of interpersonal relations and closeness among group members have shown to have implications in the collective processes experienced by athletes and non-professional practitioners during sports and physical activities. Hence, when shared flow and emotional synchrony are experienced among group members perceived efficacy as a group increases and consequently their performance. By exploring the psychosocial factors that enhance, inhibit, or disrupt collective efficacy, practitioners of sports or other related physical activities may be able to achieve optimal experiences more frequently, which, in turn, should ultimately lead them to better outcomes. Interventions aimed at developing these psychosocial processes among group members would improve collective efficacy in sports and other physical activities. One of such interventions that can improve flow and other related positive affective states is mindfulness, a non-judgmental attentional training technique. Mindfulness involves paying attention to one's body and becoming aware of all different sensations and thoughts involved in one's actions at each moment (Aherne et al., 2011; Amutio et al., 2015b). Other examples of interventions include cognitive restructuring and relaxation, just to cite a few. The aforementioned considerations are useful for promoting subjective well-being, as well as to enhance collective efficacy and, ultimately, performance during sports training.

In sum, the results of this study indicate that identification and fusion with the group are part of the collective experience in sports, promoting perceived efficacy of team members. These findings remark the utility of collective actions and social identities to explain the psychosocial processes related to collective efficacy in physical and sports activities.

## Funding

This work was supported by the Spanish Ministry of Economy and Competitiveness [under Grant PSI2014-51923-P], the University of the Basque Country [under Grant IT-666-13, Grant US13/11, and Grant UFI 11/04]; and Basque Government's Research Personnel Education and Training Program scholarship granted to LZ [PRE_2013_1_738].

### Conflict of interest statement

The authors declare that the research was conducted in the absence of any commercial or financial relationships that could be construed as a potential conflict of interest.
